# Emerging Grounded Shared Vocabularies Between Human and Machine, Inspired by Human Language Evolution

**DOI:** 10.3389/frai.2022.886349

**Published:** 2022-04-26

**Authors:** Tom Kouwenhoven, Tessa Verhoef, Roy de Kleijn, Stephan Raaijmakers

**Affiliations:** ^1^Creative Intelligence Lab, Leiden Institute for Advanced Computer Science, Leiden University, Leiden, Netherlands; ^2^Cognitive Psychology Unit, Institute of Psychology, Leiden University, Leiden, Netherlands; ^3^TNO, Information and Communication Technology, Delft, Netherlands; ^4^Leiden University Centre for Linguistics, Leiden University, Leiden, Netherlands

**Keywords:** conversational AI, language evolution, human-machine communication, grounded vocabularies, human-machine cooperation

## 1. Introduction

Building conversational AI systems has the goal to teach machines to understand human language and respond naturally. The most common way to train agents to produce and interpret natural language is currently by exposing them to large quantities of data. Although this has resulted in advances in many areas, these systems typically have little understanding of how language is related to the real world (Mordatch and Abbeel, 2018), known as the *grounding problem*. Also, most conversational agents are trained in isolation, while humans are social animals, deeply embedded in culture and surrounded by others. Complex human behaviors, like language, evolved in socio–cultural contexts and could not exist without a variety of minds using and transmitting these behaviors.

To overcome this problem, researchers in Computational Linguistics have started modeling emerging communication setups, in which novel signals are created by interacting agents (Lazaridou et al., 2018; Mordatch and Abbeel, 2018; Chaabouni et al., 2019; ter Hoeve et al., 2021). However, the findings in such models do not always match what is found in similar experiments with humans, and features found in human language often do not emerge (Lazaridou et al., 2020).

The mechanisms that influence the emergence of communication and linguistic structure have been studied in the field of Language Evolution. Although the precise origins of human language are widely debated, computer simulations (Boer, 2006; Steels, 2012a; Kirby, 2017) and experiments in which humans use novel communication signals (Galantucci and Garrod, 2010; Scott-Phillips and Kirby, 2010; Kirby et al., 2014), have revealed some key mechanisms that drive the initial emergence of a novel language and the gradual appearance of more complex linguistic structure. We review relevant findings and propose to apply methods that confirm the importance of including micro–societies of interacting minds to the emergence of novel human–machine communication systems.

**Figure 1:**
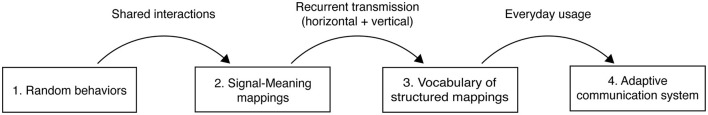
The proposed road of evolving a natural human–machine communication system. First, initially random behaviors obtain meanings and become more structured through recurrent horizontal and vertical transmission. Everyday usage facilitates the continuous evolution of communication systems which are adaptive to biases of humans and machines.

A major insight from these studies is that language *adapts* to human biases and how it is learned and used (Kirby et al., 2014, 2015). Similarly, current language models also exhibit biases, free order case-marking languages are for example more challenging to model than fixed-order languages (Bisazza et al., 2021). As such, we suggest that language used in human–machine communication should also evolve more naturally, resulting in a grounded communication system adapted to biases and constraints of human and machine learning. We moreover emphasize the importance of co–development of shared vocabularies by conversational partners (human or AI–based). Doing so might result in a dynamic communication system that is natural to humans and artificial conversational agents. We propose to follow a process of several steps, displayed in Figure 1. Starting from random behaviors, a signal–meaning mapping emerges from shared interactions (section 2) which become more structured through horizontal and vertical transmission (section 3) and eventually evolve into an adaptive communication system (sections 4, 5).

## 2. Emergence of communication

Successful communication happens when the coordinated actions of all participants adhere to the grounding criterion: that interlocutors agree that they have understood what was meant for the current purposes (Clark and Brennan, 1991). This requires a vocabulary that is (partially) aligned between interlocutors of a conversation (Pickering and Garrod, 2004). The emergence of which starts with agreeing on what kind of (initially random) behaviors should be interpreted as communicative and what they refer to (box 1 and 2 in Figure 1).

Experiments with human participants have been conducted to study the emergence of novel communication forms (Galantucci, 2005; Steels, 2006; Scott-Phillips et al., 2009; Galantucci and Garrod, 2010). Here, participants need to invent and negotiate novel signals to solve a communicative or cooperative task. Albeit often bound to the starting conditions of the experiment, even when no conventional signaling device is given, actions may gradually become communicative (Scott-Phillips et al., 2009). Typically, humans quickly establish conventions and settle on a shared set of signals. Sufficient common ground, interactions, and social coordination have been identified as crucial to facilitate the emergence of communication systems.

With computational agents, Quinn (2001) investigated the emergence of signals and cooperation without dedicated communication channels in a way that is comparable to the work of Scott-Phillips et al. (2009). Here, robots, only equipped with sensors to observe a shared environment, were tasked to move away from a starting point while maintaining proximity to each other. Initial random behaviors gradually evolved into an iconic signaling system that could establish the allocation of leader–follower roles (Quinn, 2001; Quinn et al., 2003).

A large body of work in *evolutionary language games*, as reviewed in Steels (2012b), has shown that agents without a pre–programmed language can develop a communication system from scratch. This happens in a self–organizing fashion, as alignment between agents arises from repeated interactions between individuals without central control. In the context of those experiments, Steels already proposed that robots can participate in the ongoing evolution of language and learn from human language users if there are sufficient situated interactions (Steels, 2012a). We think that this is key to developing natural communication between humans and machines.

Although building an initially shared vocabulary is well–explored between humans and in agent–based models, to the best of our knowledge, it is rarely applied in human–machine settings. One exception is a large–scale exhibition of Steel's Talking Heads experiment (Steels, 1999), in which both agents and human visitors proposed new words that could become part of an evolving shared vocabulary. We propose to revisit this idea in the context of conversational AI and include this initial step of co–developing a shared vocabulary, rather than bypassing it with (random) symbols or pre–trained language models, and trust in the process of self–organization to facilitate the emergence of conventional signal–meaning mappings. Once established, this shared set of signals may be far removed from natural language that humans know today, but just like human languages have, will adapt to their users and usage and become more complex and systematic.

## 3. Emergence of structure in language systems

Human language is uniquely structured and exhibits systematicity at multiple levels (Kirby, 2017). For example, words are combined into sentences such that their meaning is a function of the meanings of the parts and the way they are combined (compositional structure). The origins of this and other types of structure have been studied using computer models and artificial language learning experiments with humans (Kirby, 2017).

Among others, two processes have been found to contribute to the emergence of structure in language (box 2 and 3 in Figure 1). The first is *cumulative cultural evolution* where (cultural) information, such as ideas or linguistic signals, is transmitted vertically along generations. An influential experiment was conducted by Kirby et al. (2008). Here, the first participant was asked to learn an artificial language and describe images with the acquired words. Subsequent participants learned the output of the previous participant. Through this process, the words gradually changed and became more compositional and learnable. Such results consistently show that increases in learnability and structure arise because languages adapt to human inductive biases to be transmitted faithfully (Griffiths and Kalish, 2007). Words and patterns that are not easily learned or interpreted, will not be reproduced by the next generation and since structured languages are more easily compressible (Kirby et al., 2015; Tamariz and Kirby, 2015), this eventually results in more learnable and structured languages.

A second process contributing to the emergence of structure in human language is *horizontal transmission*. Here, linguistic structure originates and evolves from social coordination through repeated interactions between individuals in micro-societies. While interactions between dyads can lead to shared vocabularies and initial regularities (Theisen-White et al., 2011; Verhoef et al., 2016), a community of users seems to be necessary for the emergence of system-wide compositional structure and efficient coding (Fay et al., 2008; Raviv et al., 2019). In these cases, pressures such as the number of interaction partners and expanding meaning spaces cause initially random languages to become more structured over time.

The effects of horizontal and vertical transmission have also been demonstrated with agent–based computer simulations (Steels and Loetzsch, 2012; Kirby, 2017). Altogether, there is overwhelming evidence suggesting that transmission (vertical or horizontal) of signals within communities contributes to the emergence of structure in language. In fact, it has been argued that both types of transmission are necessary to get a language that is learnable and usable (Kirby et al., 2015). These processes should therefore be projected onto the human–machine language evolution scenario to evolve a vocabulary that shares features with human language and is equally adapted to be learned and used by machines.

## 4. Human–machine language evolution and reinforcement learning

Recent work in Computational Linguistics started to train machines to understand human language through the emergence of communication systems (Lazaridou et al., 2017; Clark et al., 2019; Manning et al., 2020). A range of work has shown that (multi–agent) reinforcement learning (RL; Sutton and Barto, 2018) can converge on communication protocols in various game scenarios (Lazaridou et al., 2016; Havrylov and Titov, 2017; Chaabouni et al., 2020). While communicative systems emerge, these often suffer from interpretability issues for humans (e.g., Mordatch and Abbeel, 2018), making its applicability to human–machine communication less obvious. The emergent protocols also often do not bear core properties of natural language (Kottur et al., 2017; Chaabouni et al., 2019). As such Lazaridou et al. (2020) use a pre–trained language model in combination with self–play to teach RL agents to communicate in natural language. However, without human intervention, this approach suffers from language drift, ultimately causing misunderstandings. While too much is problematic, we argue that some language drift is welcome since it can result in language that is optimized for human–machine communication.

A growing trend in RL advocates to include human feedback in the learning loop to improve learning (Arzate Cruz and Igarashi, 2020; ter Hoeve et al., 2021). Bignold et al. (2021) propose *assisted reinforcement learning* where information external from the environment is used to improve the performance of a learner agent and scale to more complex scenarios. Human feedback can, for example, directly be included in the behavior of an agent instead of learning it from the ground up and potentially prevents too much language drift. This draws parallels to human interactions which offer a means to ground signs through recurrent and reciprocal usage (Garrod et al., 2007), provide feedback on the success of a conversational contribution, and alleviate miscommunications resulting from partially aligned vocabularies due to variations or dialects.

To establish mutual understanding, we propose to use assisted reinforcement learning and revisit signaling games (Scott-Phillips et al., 2009), referential games (Steels and Loetzsch, 2012; Chaabouni et al., 2020), and navigation games (Mordatch and Abbeel, 2018; Dubova and Moskvichev, 2020) to evolve shared vocabularies between humans and machines. The next step is not only to use more complex problems (e.g., increasing the number of objects, interacting partners, or vocabulary size) that necessitate more complex syntax and vocabularies (Mordatch and Abbeel, 2018) but also to continue interacting with conversational agents frequently (box 3 and 4 in Figure 1). While communities of self–playing RL agents interact and consolidate the learned behaviors, frequent human–agent interactions prevent too much language drift. The evolved communication systems will not take the same form as human language initially, but through iterations, may come closer toward it and evolve into a form that makes human–machine interactions more natural, with communication systems adapted to biases in both human and machine learning.

We are aware that this proposition poses challenges, for example, the tutoring role and interactions that humans must supply. However, we assume that humans are willing to do so since we perceive robots as social, communicative partners (Guzman and Lewis, 2020) and linguistically align to computers in several ways (Branigan et al., 2010), an effect that is even stronger when computers themselves also exhibit alignment behavior (Spillner and Wenig, 2021).

## 5. Conclusion

This article proposed to combine insights from human language evolution, specifically concerning the influence of vertical and horizontal transmission, with assisted reinforcement learning. We have shown how signals and structure emerge in socio–cultural contexts and that language adapts to how it is learned and used. We therefore suggest that language used in human–machine communication should also evolve naturally, emphasizing the importance of co–development of shared conventions during communication. A first step would be to revisit communicative games and evolve successful systems between humans and machines. Doing so allows communication systems to adapt to the biases of both parties while mutual understanding is maintained, ultimately benefiting the communicative capacity of conversational agents.

## Author Contributions

This opinion piece is written by TK with help of TV. TK, TV, and RK: literature research was conducted. TK, TV, RK, and SR: reviewing and editing was done. TV and SR supervised the process. All authors contributed to the article and approved the submitted version.

## Conflict of Interest

SR is employed by TNO. The remaining authors declare that the research was conducted in the absence of any commercial or financial relationships that could be construed as a potential conflict of interest.

## Publisher's Note

All claims expressed in this article are solely those of the authors and do not necessarily represent those of their affiliated organizations, or those of the publisher, the editors and the reviewers. Any product that may be evaluated in this article, or claim that may be made by its manufacturer, is not guaranteed or endorsed by the publisher.

## References

[B1] Arzate Cruz C. Igarashi T. (2020). “A survey on interactive reinforcement learning: design principles and open challenges,” in Proceedings of the 2020 ACM Designing Interactive Systems Conference, 1195–1209.

[B2] Bignold A. Cruz F. Taylor M. E. Brys T. Dazeley R. Vamplew P.. (2021). A conceptual framework for externally-influenced agents: an assisted reinforcement learning review. J. Ambient Intell. Humanized Comput. 1–24. 10.1007/s12652-021-03489-y

[B3] Bisazza A. Üstün A. Sportel S. (2021). On the difficulty of translating free-order case-marking languages. Trans. Assoc. Comput. Linguist. 9, 1233–1248. 10.1162/tacl_a_00424

[B4] Boer B. d. (2006). “Computer modelling as a tool for understanding language evolution,” in Evolutionary Epistemology, Language and Culture (Springer), 381–406.

[B5] Branigan H. P. Pickering M. J. Pearson J. McLean J. F. (2010). Linguistic alignment between people and computers. J. Pragmat. 42, 2355–2368. 10.1016/j.pragma.2009.12.012

[B6] Chaabouni R. Kharitonov E. Bouchacourt D. Dupoux E. Baroni M. (2020). Compositionality and generalization in emergent languages. arXiv preprint arXiv:2004.09124. 10.18653/v1/2020.acl-main.407

[B7] Chaabouni R. Kharitonov E. Dupoux E. Baroni M. (2019). Anti-efficient encoding in emergent communication. Adv. Neural Inf. Process. Syst. 32.

[B8] Clark H. H. Brennan S. E. (1991). Grounding in communication.

[B9] Clark K. Khandelwal U. Levy O. Manning C. D. (2019). What does bert look at? an analysis of bert's attention. arXiv preprint arXiv:1906.04341. 10.18653/v1/W19-4828

[B10] Dubova M. Moskvichev A. (2020). “Effects of supervision, population size, and self-play on multi-agent reinforcement learning to communicate,” in ALIFE 2020: The 2020 Conference on Artificial Life (Cambridge, MA: MIT Press), 678–686.

[B11] Fay N. Garrod S. Roberts L. (2008). The fitness and functionality of culturally evolved communication systems. Philos. Trans. R. Soc. B Biol. Sci. 363, 3553–3561. 10.1098/rstb.2008.0130 18799421 PMC2607338

[B12] Galantucci B. (2005). An experimental study of the emergence of human communication systems. Cogn. Sci. 29, 737–767. 10.1207/s15516709cog0000_34 21702792

[B13] Galantucci B. Garrod S. (2010). Experimental semiotics: a new approach for studying the emergence and the evolution of human communication. Interact. Stud. 11, 1–13. 10.1075/is.11.1.01gal

[B14] Garrod S. Fay N. Lee J. Oberlander J. MacLeod T. (2007). Foundations of representation: where might graphical symbol systems come from? Cogn. Sci. 31, 961–987. 10.1080/03640210701703659 21635324

[B15] Griffiths T. L. Kalish M. L. (2007). Language evolution by iterated learning with bayesian agents. Cogn. Sci. 31, 441–480. 10.1080/15326900701326576 21635304

[B16] Guzman A. L. Lewis S. C. (2020). Artificial intelligence and communication: a human-machine communication research agenda. New Media Soc. 22, 70–86. 10.1177/1461444819858691

[B17] Havrylov S. Titov I. (2017). Emergence of language with multi-agent games: learning to communicate with sequences of symbols. Adv. Neural Inf. Process. Syst. 30.

[B18] Kirby S. (2017). Culture and biology in the origins of linguistic structure. Psychon. Bull. Rev. 24, 118–137. 10.3758/s13423-016-1166-7 28120320 PMC5325872

[B19] Kirby S. Cornish H. Smith K. (2008). Cumulative cultural evolution in the laboratory: an experimental approach to the origins of structure in human language. Proc. Natl. Acad. Sci. U.S.A. 105, 10681–10686. 10.1073/pnas.0707835105 18667697 PMC2504810

[B20] Kirby S. Griffiths T. Smith K. (2014). Iterated learning and the evolution of language. Curr. Opin. Neurobiol. 28, 108–114. 10.1016/j.conb.2014.07.014 25062470

[B21] Kirby S. Tamariz M. Cornish H. Smith K. (2015). Compression and communication in the cultural evolution of linguistic structure. Cognition 141, 87–102. 10.1016/j.cognition.2015.03.016 25966840

[B22] Kottur S. Moura J. M. Lee S. Batra D. (2017). Natural language does not emerge'naturally'in multi-agent dialog. arXiv preprint arXiv:1706.08502. 10.18653/v1/D17-1321

[B23] Lazaridou A. Hermann K. M. Tuyls K. Clark S. (2018). Emergence of linguistic communication from referential games with symbolic and pixel input. arXiv preprint arXiv:1804.03984.

[B24] Lazaridou A. Peysakhovich A. Baroni M. (2017). Multi-agent cooperation and the emergence of (natural) language. arXiv preprint arXiv:1612.07182.

[B25] Lazaridou A. Pham N. T. Baroni M. (2016). Towards multi-agent communication-based language learning. arXiv preprint arXiv:1605.07133.

[B26] Lazaridou A. Potapenko A. Tieleman O. (2020). Multi-agent communication meets natural language: synergies between functional and structural language learning. arXiv preprint arXiv:2005.07064. 10.18653/v1/2020.acl-main.685

[B27] Manning C. D. Clark K. Hewitt J. Khandelwal U. Levy O. (2020). Emergent linguistic structure in artificial neural networks trained by self-supervision. Proc. Natl. Acad. Sci. U.S.A. 117, 30046–30054. 10.1073/pnas.1907367117 32493748 PMC7720155

[B28] Mordatch I. Abbeel P. (2018). “Emergence of grounded compositional language in multi-agent populations,” in Proceedings of the AAAI Conference on Artificial Intelligence, Vol. 32.

[B29] Pickering M. J. Garrod S. (2004). Toward a mechanistic psychology of dialogue. Behav. Brain Sci. 27, 169–190. 10.1017/S0140525X04000056 15595235

[B30] Quinn M. (2001). “Evolving communication without dedicated communication channels,” in European Conference on Artificial Life (Springer), 357–366.

[B31] Quinn M. Smith L. Mayley G. Husbands P. (2003). Evolving controllers for a homogeneous system of physical robots: Structured cooperation with minimal sensors. Philos. Trans. R. Soc. Lond. A Math. Phys. Eng. Sci. 361, 2321–2343. 10.1098/rsta.2003.1258 14599322

[B32] Raviv L. Meyer A. Lev-Ari S. (2019). Compositional structure can emerge without generational transmission. Cognition 182, 151–164. 10.1016/j.cognition.2018.09.010 30267952

[B33] Scott-Phillips T. C. Kirby S. (2010). Language evolution in the laboratory. Trends Cogn. Sci. 14, 411–417. 10.1016/j.tics.2010.06.006 20675183

[B34] Scott-Phillips T. C. Kirby S. Ritchie G. R. (2009). Signalling signalhood and the emergence of communication. Cognition 113, 226–233. 10.1016/j.cognition.2009.08.009 19740461

[B35] Spillner L. Wenig N. (2021). “Talk to me on my level-linguistic alignment for chatbots,” in Proceedings of the 23rd International Conference on Mobile Human-Computer Interaction, 1–12.

[B36] Steels L. (1999). The Talking Heads Experiment. Vol. 1. Words and Meanings. Special pre-print for the LABORATORIUM Antwerpen.

[B37] Steels L. (2006). Experiments on the emergence of human communication. Trends Cogn. Sci. 10, 347–349. 10.1016/j.tics.2006.06.002 16828573

[B38] Steels L. (2012a). “Grounding language through evolutionary language games,” in Language Grounding in Robots (Springer), 1–22.

[B39] Steels L. (2012b). “Self-organization and selection in cultural language evolution,” in Experiments in Cultural Language Evolution (John Benjamins), 1–37.

[B40] Steels L. Loetzsch M. (2012). The grounded naming game. Exp. Cult. Lang. Evolut. 3, 41–59. 10.1075/ais.3.04ste

[B41] Sutton R. S. Barto A. G. (2018). Reinforcement Learning: An Introduction. MIT Press.

[B42] Tamariz M. Kirby S. (2015). Culture: copying, compression, and conventionality. Cogn. Sci. 39, 171–183. 10.1111/cogs.12144 25039798

[B43] ter Hoeve M. Kharitonov E. Hupkes D. Dupoux E. (2021). Towards interactive language modeling. arXiv preprint arXiv:2112.11911.

[B44] Theisen-White C. Kirby S. Oberlander J. (2011). “Integrating the horizontal and vertical cultural transmission of novel communication systems,” in Proceedings of the Annual Meeting of the Cognitive Science Society, Vol. 33, 956–961.

[B45] Verhoef T. Walker E. Marghetis T. (2016). “Cognitive biases and social coordination in the emergence of temporal language,” in Proceedings of the 38th Annual Conference of the Cognitive Science Society (Austin, TX: Cognitive Science Society), 2615–2620.

